# eNAL++: a new and effective off‐line correction protocol for rotational setup errors when using a robotic couch

**DOI:** 10.1120/jacmp.v16i6.5583

**Published:** 2015-11-08

**Authors:** Daan Martens, Mark Luesink, Henk Huizenga, Kasper L. Pasma

**Affiliations:** ^1^ Department of Radiation Oncology Radboud University Medical Centre Nijmegen The Netherlands; ^2^ Physics Department Radiotherapy Group Arnhem Arnhem The Netherlands

**Keywords:** robotic couch, correction protocol, rotational errors

## Abstract

Cone‐beam CTs (CBCTs) installed on a linear accelerator can be used to provide fast and accurate automatic six degrees of freedom (6DoF) vector displacement information of the patient position just prior to radiotherapy. These displacement corrections can be made with 6DoF couches, which are primarily used for patient setup correction during stereotactic treatments. When position corrections are performed daily prior to treatment, the correction is deemed "online". However, the interface between the first generation 6DoF couches and the imaging software is suboptimal. The system requires the user to select manually the patient and type the match result by hand. The introduction of 6DoF setup correction for treatments, other than stereotactic radiotherapy, is hindered by both the high workload associated with the online protocol and the interface issues. For these reasons, we developed software that fully integrates the 6DoF couch with the linear accelerator. To further reduce both the workload and imaging dose, three off‐line 6DoF correction protocols were analyzed. While the protocols require significantly less imaging, the analysis assessed their ability to reduce the systematic rotation setup correction. CBCT scans were acquired for 19 patients with intracranial meningioma. The total number of CBCT scans was 856, acquired before and after radiotherapy treatment fractions. The patient positions were corrected online using a 6DoF robotic couch. The effects on the residual rotational setup error for three off‐line protocols were simulated. The three protocols used were two known off‐line protocols, the no action level (NAL) and the extended no action level (eNAL), and one new off‐line protocol (eNAL++). The residual setup errors were compared using the systematic and random components of the total setup error. The reduction of the rotational setup error of these protocols was optimized with respect to the required workload (i.e., number of CBCTs required). Rotational errors up to 3.2° were found after initial patient setup. The eNAL++ protocol achieved a reduction of the systematic rotational setup error similar to that of the online protocol (pitch from 0.8° to 0.3°), while requiring 70% fewer CBCTs. With a 6DoF robotic couch, translation, and rotation patient position corrections can be performed off‐line to reduce the systematic setup error, workload, and patient scan dose.

PACS numbers: 87.56.Fc, 87.56.Da, 87.57.‐s

## I. INTRODUCTION

Image‐guided radiotherapy (IGRT) using cone‐beam CT (CBCT) for position verification has become widely available. It provides fast and accurate automatic vector displacement information of the patient position just prior to treatment with six degrees of freedom (6DoF, three translations and three rotations). As a consequence of this CBCT imaging, it has become apparent that sometimes significant corrections for rotations are required.^1^, ^2^, ^3^, ^4^, ^5^, ^6^

Since most treatment couches only allow translational corrections, it is still clinical practice to correct the patient position using only translations; in some cases partial rotation information is incorporated in the calculated correction. The full rotational information available from CBCT imaging is not used.

Neglecting a typical rotational setup error of 2° may cause up to 1.7 mm deviation at a distance of 50 mm from the isocenter. This can occur when the planning target volume (PTV) or an organ at risk (OAR) is located off‐axis, or if the PTV is not shaped spherically. In the patient group investigated in this study, rotational errors up to 3.2° were observed, in spite of the mask fixation.

These deviations can be relevant, especially when treatments include use of the latest generation multileaf collimators (MLCs) that allow interdigitation. With these MLCs, multiple lesions can be irradiated with a single beam segment using one centrally located isocenter. The dose distribution delivered can significantly deviate from the planned dose distribution if a rotation occurs.^1^, ^2^, ^3^, ^4^ The deviation can only be avoided or reduced by using one isocenter per lesion. This treatment is time‐consuming if there are more than two to three lesions. A study by Teng et al.^(7)^ focused on the effect of rotational setup errors in intracranial stereotactic radiotherapy. They performed a gamma analysis using 3% and 3 mm as dose difference and distance‐to‐agreement criteria, and calculated the passing ratio ≥95%. The study showed that translational errors of 1.5 mm and rotational errors of 1° resulted in a passing ratio of 62.2%.^(7)^ Furthermore, underdosage due to rotations has been found in cases of spinal radiosurgery.^(5)^ For a case where an OAR was located off‐axis, namely the spinal cord, a rotational error of 3° to 5° resulted in an increase in maximum spinal cord dose of 3.1% to 6.4%.^(6)^

In the last decade, robotic table tops have become available that allow 6DoF corrections. These 6DoF couches are primarily used for patient setup correction during stereotactic treatments. Daily imaging is required for these treatments and the resulting position correction is performed prior to treatment (online). However, the interface between the first generation 6DoF couches and the IGRT software is suboptimal. During clinical practice, the patient ID number must be manually selected and the 6DoF vector resulting from the match in the IGRT software must be manually entered. This lack of integration requires additional CBCTs to verify the result of the online repositioning, to prevent mispositioning due to manual data entry (e.g., typing) errors.

The high workload associated with the online protocol, and the interface issues, hinder the introduction of 6DoF setup corrections for treatments other than stereotactic radiotherapy. Therefore, we developed software that fully integrates a 6DoF couch with a linear accelerator. To further reduce the workload, three off‐line 6DoF correction protocols were analyzed. They require 70%–90% fewer CBCT scans per patient, since they do not require daily imaging. Off‐line correction protocols are widely used to correct for the systematic translation setup errors. Examples of well‐known off‐line correction protocols are "no action level" (NAL),^(8)^ "shrinking action level" (SAL),^(9)^ and "extended no action level" (eNAL).^(10)^ Despite drastically reducing the number of CBCTs required, these correction protocols still manage to reduce the systematic setup error. The systematic error has the biggest impact on the margin that is needed for accurate dose coverage of the tumor.^(11)^ Hence, these off‐line protocols provide a valid alternative to an online protocol, and have the benefit of reducing the workload associated with patient positioning. Furthermore, fewer CBCTs reduce the dose to the patient from imaging. For head‐and‐neck protocols, this imaging dose is ~ 1 mGy/fraction. For chest and pelvis protocols, the imaging dose per fraction is ~ 17 and ~ 24 mGy/fraction, respectively.^(12)^ Limiting the number of CBCTs is therefore relevant for fractionated treatments.

In this report we present a new method for off‐line rotational corrections. Off‐line protocols for translational setup errors have been well investigated.^9^, ^10^, ^13^ Furthermore, the effect of rotational setup errors on the planned dose distribution is also well known for various treatment sites.^1^, ^2^, ^3^, ^4^, ^5^, ^6^ Consequently, the focus of this report was off‐line correction protocols for rotational setup errors. The protocols were tested using a clinical patient database, in which all patients were treated with a 6DoF robotic couch.

## II. MATERIALS AND METHODS

The rotational setup error was measured for a group of clinical patients. The resulting database was used to determine the ability of the off‐line protocols to reduce this measured rotational setup error, compared to the online procedure. Pitch, roll, and yaw are used in this report for describing rotations around the x‐axis, y‐axis, and z‐axis, respectively, as defined in the IEC 61217 standard.^(14)^

### A. Hardware

All data were acquired at the Institute for Radiation Oncology, Arnhem, The Netherlands. Patients were treated using two Elekta linear accelerators (Elekta Oncology Systems Ltd, Crawley, UK), both equipped with the Synergy X‐ray Volume Imaging (XVI) system to acquire and process the CBCT scans. Using the XVI software (v4.5), an automatic grey value mask registration was performed, resulting in a 6DoF correction vector.

The 6DoF correction was performed using the HexaPOD treatment table from Medical Intelligence (Schwabmünchen, Germany) with the iGUIDE v1.0 software on one linear accelerator and the Protura 6D couch (CIVCO Medical Solutions, Coralville, IA) with software version 1.2 on the other linear accelerator. According to the manufacture's specifications, they both have a positioning accuracy smaller than 0.2 mm for translations and 0.2° for rotations. They can perform yaw, pitch, and roll corrections of up to 3°.

The iGUIDE v1.0 software used in this study requires the user to manually enter the required 6DoF vector. iGUIDE v2.0 features automatic transfer of the 6DoF vector. The Protura v1.2 software was developed in cooperation with the manufacturer to allow safe and fast 6DoF corrections by integrating the various systems. Patient selection is automatically performed by interfacing with the MOSAIQ Record and Verify system. The 6DoF vector from the Elekta XVI database is automatically imported and the linear accelerator operation is interlocked if irradiation is attempted before the 6DoF correction is completed. All treatments were delivered using the Elekta Beam Modulator MLC with a leaf width of 4 mm.

### B. Patient treatments

**Figure 1:**
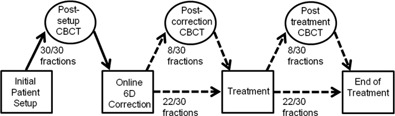
Overview of the CBCTs acquired for each treatment fraction of the 19 patients.

A total of 19 patients with intracranial meningioma were treated in 28 or 30 fractions of 2 Gy. For this patient group, a clinical target volume (CTV) to PTV margin of 2 mm was used. The patients were immobilized using a hybrid three‐point head mask without a nose hole (33740/NH) by Orfit (Orfit Industries, Antwerp, Belgium). Initially the patients were aligned using room lasers. For all fractions, a CBCT was acquired after initial setup ("post‐setup") and the 6DoF correction was applied using the 6DoF robotic couch. Furthermore, for an average of 8 treatment fractions of each patient, second and third CBCTs were obtained. The second was acquired after the setup correction was performed, prior to the treatment ("post‐correction"), and the third was acquired after the treatment ("post‐treatment"). A schematic overview of the patient workflow is depicted in Fig. 1. The post‐correction CBCTs were used to assess any residual positional error after a patient position (due to potential counter movement^(15)^ of the patient) is corrected in 6DoF. The dataset for all patients consisted of 856 CBCTs.

### C. Terminology

The terminology for describing rotational setup errors in this study is adapted from the commonly used terminology for describing translational setup errors,^(13)^ that is to say that a distinction between systematic and random setup errors is made. The population systematic setup error for rotations ($\mu_{\text{rot}}$) is the mean of the systematic setup errors of all patients. The rotational systematic setup error ($S_{\text{rot}}$) of a patient is defined as the averaged rotational setup error over all fractions of that patient. When a group of patients is considered, these systematic setup errors yield a variance that is characterized by its standard deviation ($\Sigma_{\text{rot}}$). Interfraction variation causes a variance in the rotational setup position of a patient. This variance is characterized by the standard deviation over the setup position ($\sigma_{\text{rot}}$). The standard deviation is known as the rotational random setup error.

### D. Protocols

Three off‐line correction protocols were tested for their ability to reduce the systematic and random rotational errors — random rotational errors, and $\sigma_{\text{rot}}$. These protocols were the NAL, the eNAL, and a new protocol: eNAL++.

The NAL protocol calculates the mean displacement over the first 3 treatment fractions and corrects all subsequent fractions using this mean displacement.^(8)^ For eNAL, the initial setup correction is the same, but additional weekly follow‐up measurements are performed.^(10)^ After a follow‐up measurement, a new setup correction is determined that is used for the subsequent treatment fractions. The setup correction, $C_{k}(f_{k})$), which is calculated after every measured fraction $\left(f_{k}=8,13,18,\ldots \right)$, is determined by

(1) $$C_{k}(f_{k})=-(S_{k}+a_{k}(f_{k}))$$

The constants $S_{k}$ and $a_{k}$ in this formula are derived from a linear least squares fit of data from the measured fractions. In this way, the estimation of the systematic setup error is updated after each measurement. This correction is used for the subsequent treatment fractions until a new correction, $C_{k}(f_{k})$), based on a new measured fraction, is determined. For a treatment of 30 fractions, eight CBCTs are required for this protocol.

A newly proposed protocol, eNAL++, is almost equivalent to the eNAL protocol. The difference is that on days when a CBCT is acquired, an online correction is applied instead of the off‐line correction (which resulted from previously measured fractions). For a treatment delivered in 30 fractions, the eNAL++ protocol results in eight online corrections and 22 off‐line eNAL corrections. For the first 3 fractions, an online correction is applied instead of no correction. This slightly reduces the systematic setup error, as well as the random setup error. This addition requires no extra workload since the 6DoF correction vector is automatically generated just after acquiring the CBCT and has to be determined for the off‐line procedure anyway.

**Figure 2:**
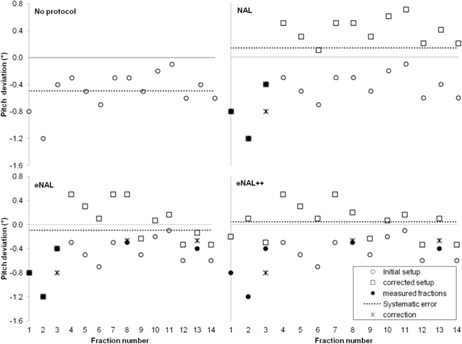
The results of NAL, eNAL, and eNAL++ protocols for the pitch rotation on the first 14 of 30 fractions of a typical patient. The open circles (○) represent the initial patient position before correction, a filled symbol (• or ▪) represents a measurement of a patient position that is used as input for the considered protocol, the open squares (□) represent the patient position after correction by the protocol. An asterisk (^*^) indicates the correction determined by the protocol. The residual systematic pitch setup error after 14 fractions for this patient is depicted with the dotted line.

Actual data from the three off‐line protocols for the first 14 fractions of an individual patient treatment course are shown in Fig. 2. All the protocols result in a reduction of the systematic error (dotted line).

### E. Comparing the protocols

The different residual errors of the four setup protocols were investigated. The residual error of an off‐line fraction was estimated by the 6DoF match result of the postsetup CBCT (see Fig. 1), combined with the 6DoF correction as calculated by the off‐line protocol. The data were available for all 28 or 30 fractions. For the online protocol and the online fractions of the eNAL++ protocol, the residual error is defined as the match result of the post‐treatment CBCT (see Fig. 1) (i.e., after the online correction). The data are available for an average of 8 of the 28 or 30 fractions.

## III. RESULTS

After the initial setup of the 19 patients using the in‐room lasers, rotational errors up to 3.2° were observed and the systematic setup error ranged from $-1.7^{\circ }\;\text{to}+1.4^{\circ }$. The results of the match between the subsequent CBCTs and the corresponding planning CT are shown in Table 1. On average, the time between the initial postsetup CBCT and the postcorrection and post‐treatment CBCTs was 4 and 15 min, respectively.

**Figure 3:**
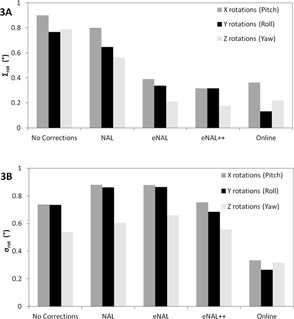
Comparison of the standard deviation (SD) of (a) systematic rotation errors ($\Sigma_{\text{rot}}$) and (b) random rotation errors ($\sigma_{\text{rot}}$) for the various correction protocols.

**Figure 4:**
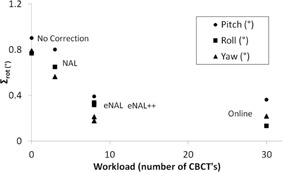
Comparison of the efficacy of the various correction protocols. The reduction in systematic error for all three rotations vs. the required workload is depicted. The patients were first positioned using a three‐point hybrid mask and aligned using the treatment room lasers. The SD of the systematic rotational error is shown for the three rotations.

In Figs. 3(a) and 3(b), the simulated effect of the off‐line protocols on the systematic and random rotational setup errors is shown. As a reference, both the uncorrected and online data are also shown. The number of required CBCTs is used as a measure for the workload. In Fig. 4, the reduction of the systematic setup error compared to the increase in workload is presented.

Of the three off‐line protocols, the NAL had the least effect on the reduction of the systematic error. The NAL also had the least workload (Fig. 3(a) and Fig. 4). The eNAL and eNAL++ protocols were similar to each other in the reduction of the systematic error. Their effect was almost equivalent to that of the online protocol, with less than a third of the workload. The small increase of the random setup error for the NAL and eNAL protocols has also been observed for translations using simulated populations.^(10)^ The eNAL++ protocol slightly reduces the random setup error, since in 8 out of the 30 fractions an online correction is applied. The workload for the eNAL++ and eNAL protocol is equal.

**Table 1:** Overview of the match results of the CBCTs with the planning CT for the 19 meningioma patients after initial setup, after correction via online imaging, and after treatment

	*Postsetup (28 or 30 fractions)*			*Postcorrection (8 fractions)*			*Post‐treatment (8 fractions)*		
	*Pitch*	*Roll*	*Yaw*	*Pitch*	*Roll*	*Yaw*	*Pitch*	*Roll*	*Yaw*
$-1.7^{\circ }\;\text{to}+1.4^{\circ }$	−0.1	−0.3	−0.2	0.0	0.0	0.0	0.1	−0.1	0.0
$\Sigma_{\text{rot}}({\;}^{\circ })$	0.9	0.8	0.8	0.2	0.3	0.2	0.4	0.1	0.2

## IV. DISCUSSION

For translations, the systematic and random setup error is included in the total systematic and random uncertainty, which partly determines the necessary CTV to PTV margin.^11^, ^15^ The impact of systematic translational errors on the margin is approximately four times larger than that of the random translational errors. The effect of rotations is not included in these margin formulae. For rotation errors, a similar effect is expected. Assume that an isocenter is located outside the PTV. A random rotational error will blur the dose distribution in the PTV. A systematic rotational error can lead to a dose distribution that does not coincide with the PTV as intended. For example, a systematic rotational error of 2° causes a 1.7 mm translation at 50 mm from the isocenter. Correction of these rotational errors allows for the CTV to PTV margin of 2 mm that was used for the patient group described here. The impact on the resulting actual dose distribution can be quantified using data from CBCT studies, as presented in this paper, combined with dose coverage simulations, which is outside the scope of this paper. However, other reports^2^, ^4^ have shown that rotational errors can have a significant effect on dose coverage. For prostate treatments, rotational corrections up to 5° are recommended when using a 3 mm margin to ensure 98% CTV coverage in more than 98% of treatments.^(16)^ When only using translational corrections, these constrains were not met. Previous research on the effect of setup errors on intracranial stereotactic radiotherapy showed that translations of 1.5 mm and rotations of 1° have a large effect on the dose distribution. Errors in such treatments should be kept below 0.7 mm and 0.5°.^(8)^ Off‐line setup protocols for rotations could reduce the residual setup errors to below this threshold, with the added advantage of a reduction in workload and imaging dose.

In this paper, we describe an effective method to minimize the rotational error, especially the most important systematic component, using a 6DoF robotic couch. Alternatively, pitch and roll errors can be minimized by adapting the collimator and gantry angles of the treatment plan during patient setup.^(17)^ Such methods have the disadvantage of compromising the clear distinction between treatment plan setup and patient setup. In clinical practice, off‐line correction protocols can be adjusted when irregular patient motion is detected. An eNAL++ protocol is easily extended by adding more than the prescribed weekly measurements. Decisions for extra measurements can be made in the time span between two fractions, thereby not hindering clinical workflow.

Previous research^(18)^ showed that, by using the HexaPOD couch and XVI imaging, online repositioning of a phantom could result in residual setup errors similar to those of the patient data presented here. Furthermore, the residual errors shown in Table 1 are small. This confirms that, when implementing our setup workflow, the introduction of a 6DoF correction protocol is not hindered by patient movement induced by table rotations. As patient movement is a concern mentioned in earlier research,^(19)^ this should be checked before implementing a correction protocol for rotations.

The next step in this research is to evaluate the eNAL++ protocol for various clinical sites, and to investigate the subsequent impact on patients' dose distributions. Treatment plans that are sensitive to rotational errors are plans with large irregular shaped PTVs or PTVs located further from the isocenter, especially if there are critical organs close to such PTVs. These plans frequently occur in our clinic. For these plans, it is interesting to investigate if they benefit from a 6DoF correction and if we can further reduce the treatment margins.

An alternative to 3D CBCT scans is orthogonal kV imaging, using two 2D images. Since orthogonal pairs have a lower dose contribution compared to CBCT, this presents a workflow that is optimized for the lowest imaging dose. Orthogonal pairs are, however, less suited for soft‐tissue imaging. Also, the same level of match accuracy cannot be expected in the presence of rotational setup errors.^(20)^

## V. CONCLUSIONS

Relatively large rotational setup errors were observed in meningioma patients, in spite of the mask fixation. The setup errors have been observed in all three rotational dimensions (pitch, roll, and yaw). Six DoF robotic couches can accurately correct these rotational setup errors. No counteracting patient motion induced by couch rotations was observed.

The off‐line eNAL++ protocol presented in this paper was shown to be effective in reducing the systematic rotational errors. The reduction was comparable to that of the gold standard, the online protocol, while requiring 70% fewer CBCT scans and hence 70% less imaging dose. In contrast to other off‐line protocols, the eNAL++ protocol also reduces the random rotation errors.

For stereotactic treatments with one or a few fractions and a high planned dose, imaging dose and workload is not an issue. For those treatments, if rotational deviations for a specific plan are an issue, an online 6DoF correction is still recommended. However, most patients are planned with more than a few fractions, which makes minimizing workload and imaging dose relevant, and off‐line 6DoF eNAL++ the recommended protocol.

## ACKNOWLEDGMENTS

We would like to thank the technologists on both linear accelerators for their continuing enthusiasm in acquiring the CBCT scans and performing the image data analysis. We would like to give a special acknowledgment for their feedback on the various hardware and software changes that were made during the development of the 6DoF workflow.
